# 

**Published:** 1880-08

**Authors:** 


### CONTENTS:

ART. I.—An Essay on Mechanical Dentistry. By Dr. W. W. Ford.................145
ART. II.—More About Rigg's Disease. By Dr. Geo. A. Mills.........................160
ART. III.—The Electro Chemical Theory. By S. B. Palmer, D. D. S...............166
ART. IV.—Treatment of Teeth with Dead Pulps and Alveolar Abscess.
By Dr. C. R. E. Koch...........171
ART. V.—The Ethics of Dentistry. By John H. Coyle, D. D. S,.....................176
ART. VI.—Washing Amalgams. By Thos. Fletcher......................................182
Virginia State Dental Association..................................................................183
American Academy of Dental Science............................................................183

### EDITORIAL, ETC.

False Teeth.....................................................................................184

### MONTHLY SUMMARY.

Recent Dental Conventions............................................................185
Influence of Tobacco Smoking upon Health.........................................186
Anæsthesia in Dentistry................................................................188
Compressed Mother-of-Pearl...........................................................188
Ready Way to Test Chloroform.........................................................189
The Tasting Power of the Tongue......................................................189
A New Anæsthetic.........................................................................189
Remedy in Thrush........................................................................190
Action of Collodion on Temperature.................................................190
Safety in Hydrobromic Ether...........................................................191
Death from Bromide Ethyl..............................................................191
American Dental Association..........................................................191
Local Anæsthesia with Bromide of Ethyl............................................192
Changes in the Maxillary B ›nes in Ataxia..........................................192
Bromide of Ethyl as a Local Anæsthetic.............................................192

